# Insanity

**Published:** 1870-11

**Authors:** 


### INSANITY

Is caused by too much blood being in the arteries of the brain ; if there is too much in the veins, it is apoplexy.

The arteries carry pure fresh blood from the heart to the brain to feed it ; the veins bring it back after it has lost its substance, its life, to be renewed in the lungs. Hence the purer the air we breathe, the more life is given to the blood that is sent to the brain, the more vigorous it becomes, the better it is nourished, the more efficiently it works, the more easily and actively it thinks, and reasons, and argues.

If a natural amount of fresh blood is sent to the brain, it acts with its natural healthful vigor ; if more than a natural amount of blood is sent there, the brighter and quicker are the thoughts.

Whatever "excites" the brain, causes an increased amount of blood to be supplied there.

In the lapse of ages, the observation of men has noted what excites the brain. Thinking excites it, draws more blood to it to feed it, as roots draw moisture to the flower to support it, to keep it alive.

Stimulants excite the brain artificially; conversation excites the brain under certain circumstances. Too much excitement, continued excitement, is disease.

If a natural, healthful amount of blood is sent to the brain through the arteries, it is naturally returned in proportionate quantities. If too much blood is sent to the brain, it will for a time return the excess, because the arteries close upon it and empty themselves.

If these arteries are made to receive a greater amount of blood than is natural and healthful, too often they begin to lose power of sending it all back, because they have been over-distended, as the bow has been overstrung, and will not contract or rebound full enough to do accustomed duty or work.

We once heard Rarey, the great Horse-Tamer, say: That if a horse fell down, in harness for example, he would try to get up; if he failed he would try again, and the third time he would try; if he did not then succeed, he would try no more; a kind of animal instinct seemed to say "It is useless."

So there is a physiological instinct in the arteries of the brain; for a few times they will try, when overfilled, to empty themselves, and do so, but if imposed upon too much, they "give it up," passively yield to the increased quantities of blood sent into them, and by degrees distend more and more: the brain is over fed; is kept feeding, taking nourishment from the blood, is stimulated all the time, and has no chance to rest, to sleep. After a while it loses the power to sleep, and that is insanity, which always comes on with increasing sleeplessness, and the first step towards recovery from it is a growing ability to sleep.

Too much business stimulates the brain, excites it, and if this is continued too long, the inevitable results are either

Insanity,
Paralysis, or
Apoplexy.

Insanity is where the brain arteries are kept too full of blood, are in a state of chronic fullness, cannot be sufficiently emptied, but there is no disorganization of machinery.

Paralysis is a loss of power; the parts have worked so much, they can work no more; paralysis generally comes on the nervous and the old, who have worked too fast, too hard, or beyond their strength; the brain being stimulated, lashes the poor body to unnatural efforts, as the exhausted, dumb brute is lashed or goaded to death, until it can move no more.

A celebrated writer died a few days ago; he left a great number of unfinished manuscripts—some with incomplete paragraphs; in others the hand had stopped in the middle of a sentence, and sometimes but half the word was completed; the stimulated brain worked on, until the instant the muscles of the hand had refused to do the bidding of the more undying part, and would be goaded no longer.

Apoplexy is where the vessels of the brain are so full, so distended, that they are ruptured; "the pitcher is broken at the fountain," and can be mended no more.

Hence it is the wisdom of all to give the brain rest from the driving stimulus of excessive, over-pressing business. Instead of keeping up the stimulus by strong excitants of coffee, and tea, and brandy, and the whole long deadly list of spirituous liquors, go and get repose in sleep, for it can only rest the brain in a natural way; and let it never be forgotten that the man who has to be kept up to the working-point by any drink or drug, is but a single inch this side of instant death. Whenever you can't do work in hand without the aid of a glass of brandy or other artificial stimulant, you are nearer the grave or the mad-house than I should like to be; a single half hour may put you in either.

